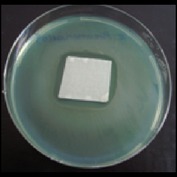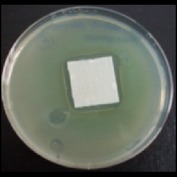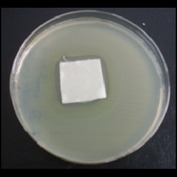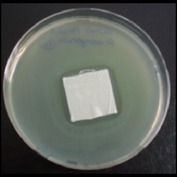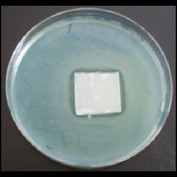# Synthesis of Silver Abietate as an Antibacterial Agent for Textile Applications

**DOI:** 10.1155/2015/215354

**Published:** 2015-02-25

**Authors:** A. Yıldız, M. Değirmencioğlu

**Affiliations:** Namık Kemal University, Corlu Engineering Faculty, 59860 Tekirdağ, Turkey

## Abstract

This study explored the potential use of new silver abietate obtained from abietic acid as an antibacterial agent for textile applications. Synthesis, structure, and antibacterial studies of silver abietate compound are reported. Silver complex was obtained reacting abietic acid with silver. The new compounds were characterized by ^1^H NMR, ^13^C NMR, DEPT, IR, UV, and ESI-MS techniques which support the proposed structures. The new Ag abietate complex has no environmental hazard, its antibacterial activities were evaluated after being applied to cotton fabric by padding process according to the JIS L 1902-2008 agar diffusion test method and against three Gram-negative and three Gram-positive bacteria, respectively. Stability of antibacterial effect after repeated washings (3, 5, 10, and 20) was also tested which indicated that the synthesized silver abietate compound could be used as a new antibacterial agent in textile industry. In this way, the compound has been synthesized the first time in the literature and the applications have been investigated.

## 1. Introduction

Abietic acid, one of the most important natural naphthenic acids, is a tricyclic diterpene. Among resin acids obtained from resins and diterpenes, the most useful acids, abietic acid (also called sylvic acid) is naturally obtained from pine resins and does not dissolve in water [1]. Several chemical substances are used for antimicrobial finishing on textiles, and finishing with metal and metal salt has an important weight among these substances. Since even low concentrations of many heavy metals are toxic for microorganisms, various metals such as copper, zinc, and cobalt have been used as an antimicrobial agent in textile industry. However, the metal most widely used for this purpose has been silver, especially in wound dresses as it has a strong antimicrobial impact, low toxicity, and little risk against bacterial resistance [2–8]. Silver ion is known to have been used since ancient times, and its impact on more than 650 pathogen microorganisms was proved in clinical experiments [9].

Silver is used in many fields of textile industry including production processes and several finished products, owing to its electrical and heat conductivity and optical reflection and antimicrobial properties [10]. Different forms of silver products used in previous studies include metallic silver [11], silver chloride [12, 13], silver zeolite [14], silver dendrimer and composites [15], polymer-silver nanoparticles [16, 17], silver nanoparticles [18–20], PU coated silver nanoparticles [21], and Ag/TiO_2_ composite nanopowders [22]. Antimicrobial fabrics are not visually different from common textile products and are produced by adding some hygienic properties.

Antimicrobial fabrics are widely used in daily life for different needs and in different places ranging from hospitals, hotels, and restaurants to baby clothes and sports clothes. Microorganisms living in textile products may damage both the product and the user. Antimicrobial textile products help to minimize or eliminate negative impacts of microorganisms by preventing the growth of infecting microorganisms as well as microorganism-induced bad smell, staining, color change, and quality loss [23].

Recently, numerous studies have been conducted to seek antibacterial agents [24–26]. This study aimed at producing, with high yield, synthesized silver abietate (abietic acid reacted with silver) so as to be used as an antibacterial agent for textile applications. Antibacterial activities of this compound were evaluated after being applied to cotton fabric by padding process [27].

## 2. Experimental

Cotton woven fabric was supplied from Denge Chemistry Co., Ltd. (Turkey). For synthesis reactions, silver nitrate (Tekkim), abietic acid (Sigma Aldrich), diethyl ether (Tekkim), and NaOH (Merck) were used.

IR spectra were taken by using a KBr table with Shimatsu IR-470 Infrared Spectrophotometer. NMR spectra were done in DMSO on a Varian Unity Inova (Varian, Palo Alto, CA, USA) 500 MHz for ^1^H NMR, ^13^C NMR, and DEPT. ESI-MS was measured on Thermo Finnigan LCQ Advantage Max LC/MS/MS apparatus (Basel, Switzerland). UV-Vis spectra of the complexes were recorded at 25°C in aqueous solution on an Agilant 4583 diode array spectrometer.

### 2.1. Synthesis of Agent

**Scheme 1:**
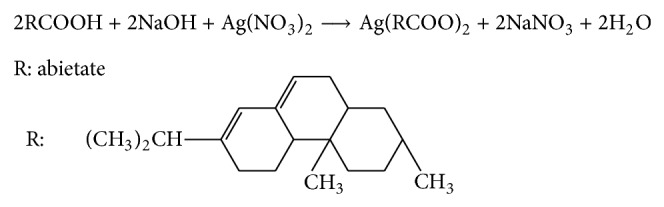


In this study, the potential use of abietate known as saturated hydrocarbons was explored as an antibacterial agent in the textile industry by following the two main steps of (1) synthesis and characterization of antibacterial agent and (2) use of synthesized antibacterial agent in textile applications. Silver abietate compound (the naphthenate silver complex) with white color was synthesized from the reaction of abietic acid with silver salt. The synthesis reaction of silver abietate compound is shown in Scheme 1.

To carry out the reaction, abietic acid was first dissolved in the organic solvent acids, and sodium salts were composed by adding caustic soda. Then silver abietate was obtained by adding silver nitrate. The synthesis of the agent was described below.

Thermometer, condenser, and dropping funnel were placed at the necks of a three-necked flask. Abietic acid solution in 10% (v) diethyl ether calculated stoichiometrically was put into the flask, and 10% (v) NaOH solution was put into the dropping funnel. The temperature was raised to 40–45°C by running a magnetic stirrer, and the solution was stirred while dropping NaOH from dropping funnel for 60 min. Medium pH was adjusted between 7 and 8. The calculated amount of 10% (v) Ag(NO_3_)_2_ solution was added to the mixture. The heater was turned on, the dropping funnel was opened, and the mixture was stirred by running an electromagnetic stirrer (Hot-Plate 300°C 15 cm circular M15 type) at room temperature for 1 h, and then this solution was held for 24 h. The solution obtained was put into the extraction flask, and liquid phase was separated from the organic phase. After the removal of solvent from the organic phase, silver abietate compound was obtained.

After the agent was synthesized, the chemical structure was analyzed with by spectroscopic techniques such as ^1^H NMR, ^13^C NMR, DEPT, IR, UV, and ESI-MS.

### 2.2. Use of Synthesized Antibacterial Agent in Textile Applications

After the synthesis of silver abietate was completed, the compound containing 40 g/L silver abietate, 1 g/L dispersing agent (Denpol HT-DengeKimya), 1 g/L wetting agent (Denwet PB 100-Denge Kimya), and acetic acid (for adjusting pH to 5) was prepared by stirring at 40–45°C for 10–15 min in an ultrasonic bath (Baysonic). Application recipe was determined by taking the pretest results into consideration. Cotton fabrics were impregnated with these liquors and dried with a tenter frame dryer at 85°C for 4 min.

Antibacterial tests were applied to the fabrics before washing and after 3, 5, 10, and 20 washings according to the JIS L 1902-2008 protocol. Test and control specimens (nonsterile) were cut into the recommended dimensions of 25 × 50 mm and sterilized by autoclaving (at 120°C for 15 min). Inoculum was prepared as follows. The bacteria were incubated for 24 h at 37 ± 2°C in nutrient broth (NB). 1.0 ± 0.1 mL from the inoculum with 1 × 10^7^ cells/mL was added to 15 mL of nutrient agar (NA) warmed at 45-46°C. This solution was disposed in a sterilized Petri dish. After agar solidification, the sterilized textile samples were placed over the agar and incubated for 24 h at 37 ± 2°C.

*Escherichia coli* (ATCC25922),* Klebsiella pneumoniae* (ATCC13883), and* Pseudomonas aeruginosa* (ATCC27853) as Gram-negative bacteria and* Staphylococcus aureus* (ATCC29213),* Bacillus subtilis* (NRRL NRS744), and* Enterococcus faecalis* (ATCC29212) as Gram-positive bacteria were used. The stability of antibacterial effect after repeated washings (3, 5, 10, and 20) was also tested according to BS EN ISO 26330 standard at 30°C where ECE Standard Detergent of SDC Enterprises, Ltd., was applied.

The structure of the synthesized compound was elucidated by spectroscopic techniques such as ^1^H NMR, ^13^C NMR, DEPT, IR, UV, and ESI-MS.

The silver abietate obtained with % 78 yield as a crystalline obtained light gray powder. The IR spectrum of silver abietate showed absorptions at 3485 (–OH), 1710 (>C=O), 1399, 1573 (carboxylic acid salt), 1630 (–C=C–), 2876, and 2941 (–CH, –CH_2_, and –CH_3_) cm^−1^. In the UV spectrum compound, maximum absorptions were observed at the 267 nm. The ^1^H NMR spectrum of the synthesized compound showed characteristic signals for a silver abietate, with the signals 0.96 (*d*, *J* = 7.2 Hz, –CH(CH_3_)) indicating the presence of two isopropyl groups and broadened singlet with singlet at *δ* 5.31 (2H, brs, H_b_) and 5.68 (2H, s, H_a_) showing unsaturated conjugated protons. The presence of two methyl groups was observed at *δ*_H_ 0.70 with a singlet signal corresponding to six protons. However, in the ^1^H NMR spectrum of compound, the singlet at *δ* 1.04 was attributed to methyl protons adjacent to C=O. In addition, in the ^1^H NMR spectrum of compound showed a multiple centered 1.07–2.11 (12 –CH_2_, 10 –CH protons).

A detailed study on the ^13^C NMR and DEPT spectra revealed 4 carbon signals due to four sp^2^ quaternary carbons, four sp^2^ methines, and two carbonyl carbons. The signal at *δ*_C_ 178.4 for the two carbonyl carbons, eight other signals of sp^2^ carbons at *δ*_C_ 1143.1, 121.2, 133.8, and 119.5, and aliphatic –CH/–CH_2_/–CH_3_ carbons at 20.8, 33.2, 23.3, 21.7, 48.9, 15.9, 42.7, 32.6, 28.7, 40.8, 34.7, 32.6, and 29.8 were characteristic of a silver abietate structure.

The silver abietate gave a molecular ion peak [M]^+^ at *m*/*z* 710.31 (15) in positive ESI-MS which was consistent with a molecular formula of C_40_H_58_O_4_Ag.

**Figure 1:**
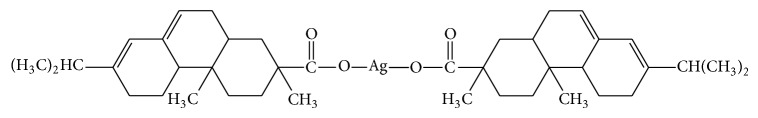
Molecular structure of silver abietate antimicrobial agent.

Based on the above evidence, the structure of compound was identified as silver abietate (Figure 1).

Antibacterial test results according to the JIS L 1902-2008 protocol standards of silver abietate compound-applied cotton fabrics before washing and after 3, 5, 10, and 20 washings are presented in Table 1.

Results of the antibacterial test for silver abietate compound-applied cotton woven fabrics before and after washings according to the JIS L 1902-2008 protocol standards are presented in Table 2.

Antibacterial activity against* B. subtilis*,* K. pneumoniae*, and* P. aeruginosa* was not different and was stronger significantly than the other bacteria. The lowest antibacterial activity was observed in* E. faecalis*. The antibacterial activities of silver abietate (silver naphthenate) applied to the cotton fabrics in successive washings were still permanent after 20 washings.

## 3. Conclusion

After silver abietate (silver naphthenate) was synthesized with the abietic acid, its use as an antibacterial agent in finishing of cotton fabrics against the three Gram-negative and three Gram-positive bacteria was found to be promising. Furthermore, the antibacterial impact was permanent even after 20 successive washings. After the washings, silver abietate was observed to exhibit a better antibacterial activity against* Bacillus subtilis, Klebsiella pneumoniae*, and* Pseudomonas aeruginosa* than the other bacteria. The antibacterial activity was strong for the three bacteria even after 20 successive washings. The lowest antibacterial activity was against* Enterococcus faecalis*. Although antibacterial activity was low against this bacterium, permanency was more important. Test results confirmed that the complex was active against the six different bacteria and was permanent after 20 successive washings. Another advantage of the obtained antibacterial agent is that the synthesized silver abietate as a complex compound cannot get onto the skin [28]. All the above considerations, together, point to silver abietate as a new antibacterial agent in textile industry. Since it has been determined in this study that silver abietate is a potential antibacterial agent, synthesis of low cost and more effective silver abietate has been planned for further work.

## Figures and Tables

**Table 1:** Inhibition zone diameters (mm) of synthesized silver abietate against Gram-positive and Gram negative bacteria measured on cotton fabric before washing and after 3, 5, 10, and 20 washings.

Bacteria	—	3rd	5th	10th	20th
*B*. *subtilis *	4	4	4	4	3
*S*. *aureus *	3	3	3	2	2
*E*. *faecalis *	2	2	2	1	0.5
*K*. *pneumoniae *	4	4	4	4	3
*E*. *coli *	3	3	3	2	1
*P*. *aeruginosa *	4	4	4	4	3

**Table 2:** Antibacterial activity of silver abietate applied cotton fabrics after 3–20 washings against six different bacteria.

Bacteria types	—	3st	5th	10th	20th
*B. subtilis *	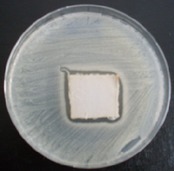	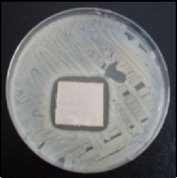	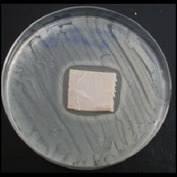	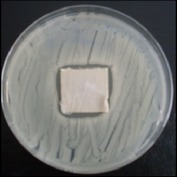	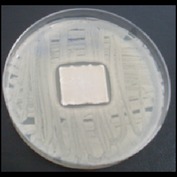

*S. aureus *	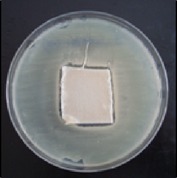	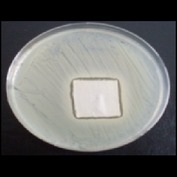	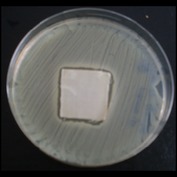	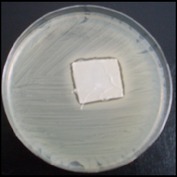	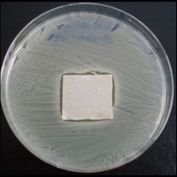

*E. faecalis *	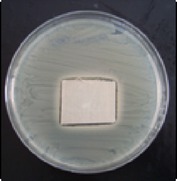	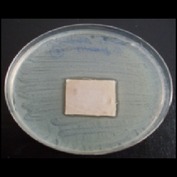	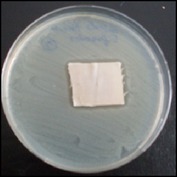	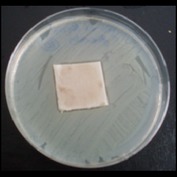	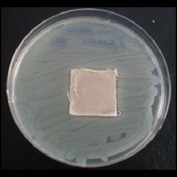

*K*. *pneumoniae *	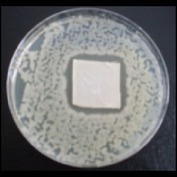	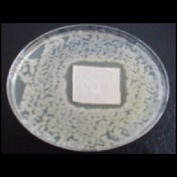	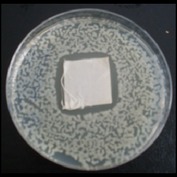	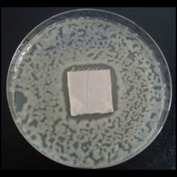	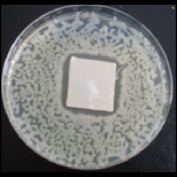

*E. coli *	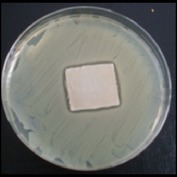	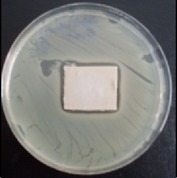	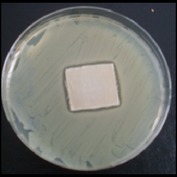	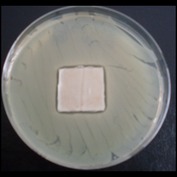	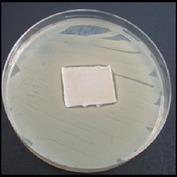

*P*. *aeruginosa *